# Extracellular vesicles for abdominal aortic aneurysm: mechanisms, therapeutic potential, and translational challenges

**DOI:** 10.3389/ebm.2026.11246

**Published:** 2026-08-28

**Authors:** Huibo Ma, Zongyou Xie, Yihang Cai, Fangda Li, Jianqiang Wu, Yuehong Zheng

**Affiliations:** 1 State Key Laboratory of Complex Severe and Rare Disease, Department of Vascular Surgery, Peking Union Medical College Hospital, Chinese Academy of Medical Sciences and Peking Union Medical College, Beijing, China; 2 National Infrastructure for Translational Medicine, Institute of Clinical Medicine, Peking Union Medical College Hospital, Chinese Academy of Medical Sciences and Peking Union Medical College, Beijing, China

**Keywords:** abdominal aortic aneurysm, cell-free therapy, engineered EVs, extracellular vesicles, precision medicine, translational challenges

## Abstract

Abdominal aortic aneurysm (AAA) is a progressive and potentially fatal vascular disease for which no effective pharmacological therapy is currently available. While surgical repair remains the only definitive treatment for advanced aneurysms, patients with small AAAs are mainly managed by surveillance, highlighting the need for disease-modifying strategies. Extracellular vesicles (EVs) have emerged as promising cell-free therapeutic tools because of their biocompatibility, ability to transfer bioactive cargo, and capacity to regulate multiple pathological processes involved in AAA progression. This review summarizes recent advances in EV-based therapies for AAA, focusing on mesenchymal stromal cell-derived EVs, immune cell-derived EVs, and engineered EV platforms. Preclinical studies suggest that therapeutic EVs can attenuate aneurysm formation by suppressing macrophage-driven inflammation, regulating macrophage polarization, inhibiting neutrophil extracellular trap-associated injury, protecting vascular smooth muscle cells from senescence, ferroptosis, apoptosis, and mitochondrial dysfunction, and limiting extracellular matrix degradation. Engineered EVs, including cargo-enriched, peptide-targeted, magnetically guided, chemotaxis-enabled, and biomaterial-assisted systems, may further improve lesion targeting, vascular retention, and therapeutic potency. However, EV-based AAA therapy remains at an early preclinical stage. Key barriers include unclear biodistribution and clearance, insufficient evidence of lesion-specific target engagement, heterogeneous EV isolation and characterization methods, uncertain dosing strategies, and the need for standardized potency, safety, manufacturing, and regulatory frameworks. Overall, EVs offer a biologically rational platform for non-surgical AAA therapy, but clinical translation requires rigorous standardization and robust evidence linking EV delivery to vascular repair.

## Impact statement

Our review addresses this unmet need by providing an updated and integrative overview of EV biology in AAA, with particular emphasis on three aspects. First, we summarize how EVs participate in AAA pathogenesis through inflammatory amplification, macrophage polarization, vascular smooth muscle cell dysfunction, extracellular matrix remodeling, ferroptosis, pyroptosis, and other lesion-relevant processes. Second, we highlight recent progress in therapeutic EVs and engineered EV platforms, including cargo-enriched EVs, peptide-targeted EVs, magnetically guided systems, chemotaxis-enabled delivery, and biomaterial-assisted retention strategies. These approaches may help overcome key limitations of conventional EV application, such as insufficient lesion targeting, rapid systemic clearance, and limited vascular wall retention. Third, we provide a detailed and balanced discussion of the unresolved controversies and translational barriers in the EV field, including evidence attribution, co-isolated non-vesicular contaminants, unclear biodistribution and clearance, insufficient lesion-level target engagement, heterogeneous isolation and characterization methods, uncertain dosing strategies, quality and safety requirements, manufacturing scalability, and regulatory readiness.

## Introduction

Abdominal aortic aneurysm (AAA) is a serious cardiovascular disease characterized by localized dilation of the abdominal aorta, with a diameter exceeding 30 mm or 50% of the normal aortic diameter [1]. AAA predominantly affects older adults, and its incidence rises markedly with age, with a prevalence of approximately 5%–10% in men over 65 years [2, 3]. Aneurysm rupture is the most feared complication, associated with very high mortality and an estimated 150,000–200,000 deaths annually worldwide [4–6]. With ongoing population aging, the proportion of individuals older than 65 years is projected to increase from 12% to 22% over the next 3 decades [7], further amplifying the public health burden of AAA and the need for improved management strategies.

Despite advances in imaging surveillance and perioperative care, AAA management continues to face major challenges. Most AAAs remain clinically silent until late-stage enlargement or rupture, and the mechanisms underlying disease initiation and progression are incompletely understood, involving chronic inflammation, vascular smooth muscle cell (VSMC) dysfunction and loss, and extracellular matrix (ECM) degradation [8]. For larger aneurysms, definitive treatment relies on open surgical repair or endovascular aortic repair (EVAR), which are invasive and costly [9]. Moreover, postoperative complications and continued aneurysm-related risks (e.g., aortic expansion or endoleak) necessitate long-term follow-up, and patients who do not meet surgical thresholds (symptomatic AAA and asymptomatic aneurysms ≥55 mm in men and ≥50 mm in women) often require prolonged surveillance and ongoing risk assessment [10]. Collectively, these realities highlight the urgent need for effective, non-surgical strategies capable of slowing or halting AAA progression.

In this context, extracellular vesicles (EVs) have gained increasing attention as a translational research focus in AAA. EVs are nanoscale, membrane-enclosed particles secreted by cells that carry diverse bioactive cargoes and participate in intercellular communication. Accumulating studies suggest that EVs are involved in shaping the AAA microenvironment and may be leveraged as therapeutic tools, including as natural or engineered delivery platforms to modulate key pathogenic processes [11–13]. Over the past 5 years, several reviews have summarized EV-related findings in AAA across pathophysiology and therapeutic development. However, there remains a notable lack of comprehensive discussion regarding the therapeutic roles of EVs in AAA, particularly with respect to the research advances and translational potential of engineered EVs in this disease. Building on this foundation, the present review provides an updated synthesis of EV biology in AAA, integrates recent advances in EV engineering and delivery concepts, and consolidates the key challenges that must be addressed for clinical translation.

## Biological characteristics and functions of EVs

### EV classification and cargoes

EVs are nanoscale vesicles with a lipid bilayer membrane secreted by cells, and they are widely present in various body fluids of organisms. In terms of size, origin and release mechanism, EVs can be divided into three categories: exosomes, microvesicles, and apoptotic bodies. Exosomes are usually between 30 and 150 nm in diameter and are formed mainly through endocytosis and released into the extracellular space through the fusion of multivesicular bodies with the plasma membrane. Microvesicles have a larger diameter, usually between 100 and 1,000 nm, and are formed by direct outward budding from the cell membrane. Apoptotic bodies are the largest vesicles among the EV types formed during the late stages of apoptosis, with diameters ranging from 500 to 5,000 nm [14]. The cargo composition of EVs is complex, including bioactive molecules such as proteins, lipids, metabolites, RNA, and DNA, which can transmit information from donor cells to recipient cells and regulate various physiological and pathological processes [15]. Currently, more than 3,000 protein species have been identified in EVs, and EVs also carry enzymes, growth factors, signaling molecules, and adhesion proteins specific to donor cells. The lipid composition of EVs mainly includes cholesterol, sphingomyelin, phosphatidylserine, and ceramide. The main RNA components in EVs include messenger RNA (mRNA) and fragments, microRNA (miRNA), long noncoding RNA (lncRNA), circular RNA (circRNA), and transfer RNA (tRNA). Recent studies have also shown that certain subpopulations of EVs contain mitochondrial components (such as mitochondrial DNA and proteins) and may play a role in repairing mitochondrial function [16]. The cargo contents are dependent on the source cell type and its functional state. The bioactive substances of EVs can not only participate in local intercellular communication but also facilitate long-distance interorgan communication through the blood circulation [17].

### EV biogenesis

The biogenesis of EVs is a complex and highly regulated process [18]. Exosome biogenesis involves the invagination of the cell membrane to form early endosomes, which further invaginate to generate multivesicular bodies (MVBs) containing intraluminal vesicles (ILVs). Finally, the vesicles within the MVBs are released into the extracellular space by fusion with the cell membrane [19]. The formation of exosomes depends mainly on the endocytic pathway within the cell in an endosomal sorting complexes required for transport (ESCRT)-dependent manner (ESCRT complex) or an ESCR-independent manner (ceramide and tetraspanin family proteins) [20, 21]. Microvesicles are formed directly by outward budding from the plasma membrane and are regulated by cytoskeleton rearrangement and membrane phospholipid redistribution [22]. Enzymes that can induce membrane curvature, calcium ion influx, calpain activation, and the RhoA GTPase signaling pathway are involved in this process. Apoptotic bodies originate from the cellular fragments of cells undergoing programmed cell death, and recent studies suggest that they play a role in immune regulation and the clearance of cellular debris [23]. The release of EVs depends on cytoskeletal components (actin and microtubules), molecular motors (dynein, kinesin, and myosin), and small GTPases [24]. The regulation of these mechanisms has a significant effect on the number, size, and composition of EVs. After EVs are secreted, they navigate the local tissue microenvironment or travel in body fluids to reach remote organs, where they interact with or enter target cells to trigger cellular phenotypic changes [25].

### EV isolation and characterization

The commonly used isolation methods for EVs include ultracentrifugation, density gradient centrifugation, ultrafiltration, size-exclusion chromatography (SEC), polymer-based precipitation, affinity capture methods, and microfluidics [26, 27]. Ultracentrifugation is a classical method for the isolation of EVs, but it requires special centrifuge equipment and is time-consuming, which may lead to the aggregation of EVs, and product purity is also affected by contamination from nonvesicular proteins. It can be combined with density gradient ultracentrifugation for further purification, and separation of different EV subpopulations. Size-based isolation methods (ultrafiltration and SEC) are relatively simple to perform, can retain EV activity, and are suitable for small-volume samples. Polymer-based precipitation is simple to perform, does not require special equipment, and is suitable for small sample volumes but may coprecipitate non-EV proteins, resulting in low purity. The affinity-based capture method can specifically separate EVs derived from a certain type of cell, but it is costly and may affect the activity of EVs. Microfluidic technology is an emerging high-throughput method for EV isolation that uses fluid dynamics or affinity principles, but this approach requires specialized equipment. These methods have their respective advantages and disadvantages, and the appropriate method should be selected according to the experimental requirements.

The commonly used characterization methods for EVs include mainly the size distribution and yield detection by nanoparticle tracking analysis (NTA) or resistive pulse sensing (TRPS); morphology detection by transmission electron microscopy (TEM); and protein marker detection by Western blot, flow cytometry, or ELISA. The positive markers of EVs include tetraspanins (CD63, CD81, and CD9), ESCRT-related proteins (TSG101 and ALIX), and heat shock proteins (HSP70 and HSP90), and the negative markers of EVs include calnexin and cytochrome C [28, 29]. The purity of EV isolation can be determined by detecting negative markers.

### EV biofunction

**FIGURE 1:**
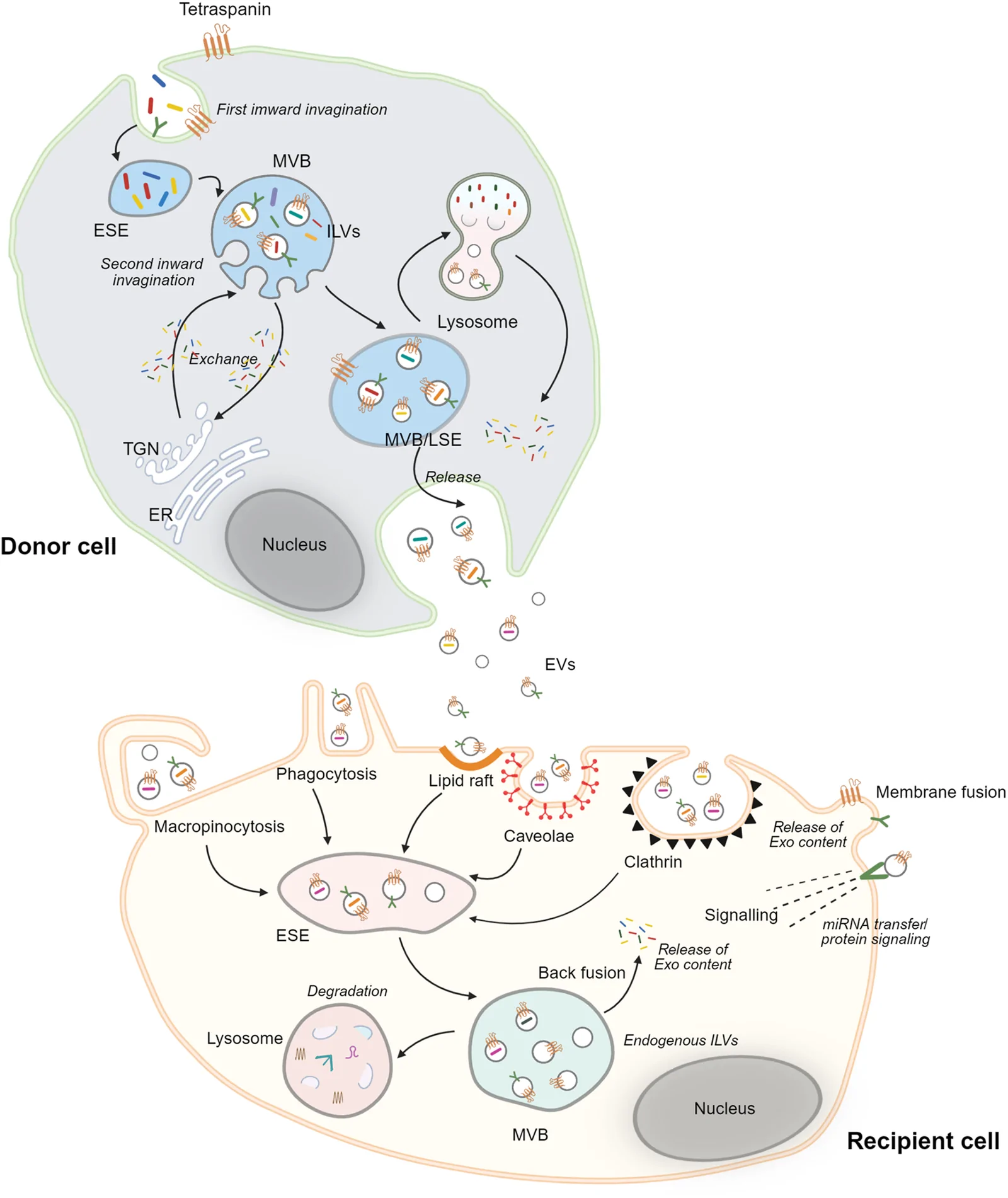
Biogenesis, release, and cellular uptake of EVs. The diagram illustrates the pathway of EV biogenesis in a donor cell, starting from endocytosis and the formation of early sorting endosomes (ESE), which mature into MVB containing ILVs. These MVBs can fuse with the plasma membrane to release exosomes into the extracellular space. Alternatively, microvesicles bud directly from the plasma membrane. The released EVs can then be taken up by a recipient cell through various mechanisms, including phagocytosis, macropinocytosis, membrane fusion, or clathrin/caveolae-mediated endocytosis, ultimately delivering their cargo to influence cell signaling and function. Key organelles involved, such as the nucleus, endoplasmic reticulum (ER), trans-Golgi network (TGN), and lysosome, are also indicated. ER, Endoplasmic Reticulum; ESE, Early Sorting Endosome; EVs, Extracellular Vesicles; ILVs, Intraluminal Vesicles; LSE, Late Sorting Endosome; MVB, Multivesicular Body; TGN, trans-Golgi Network.

EVs play an important role in intercellular communication by carrying bioactive cargo (Figure 1). This communication can be achieved through either autocrine or paracrine signaling. EVs can regulate the function and fate of neighboring or distant target cells either by interacting with the receptor ligands of target cells to trigger intracellular signaling, fusing with the plasma membrane of target cells and releasing bioactive cargo contents directly into the cytoplasm, or being internalized into target cells through endocytic pathways [30, 31]. Once EVs were regarded as cellular waste disposal units. Currently, an increasing number of studies have shown that EVs can regulate various biological functions, including gene transcription and translation, immune responses, angiogenesis, tissue repair and regeneration, receptor–ligand signaling, metabolic reprogramming, cell survival and proliferation, cell differentiation, and apoptosis [32].

Moreover, recent studies have shown that EVs participate in the regulation of vascular aging. EVs promote or delay vascular aging by regulating the senescence and function (proliferation, migration, apoptosis, differentiation, and inflammation) of vascular cells, regulating vascular calcification, and mediating the remodeling of the ECM. In addition, EVs regulate the vascular microenvironment by transmitting signaling molecules, which may be related to the pathogenesis of vascular aging-related cardiovascular diseases [33–35]. In aging-related diseases, the compositional changes of EVs are important biomarkers that can reflect the progression of vascular aging and related pathological states. Moreover, EVs derived from stem cells/progenitor cells also have antiaging and damage repair effects [36, 37]. These findings also provide new insights into EVs as potential therapeutic targets or therapeutic agents for aging-related cardiovascular diseases.

## Aortic structure and AAA microenvironment

**FIGURE 2:**
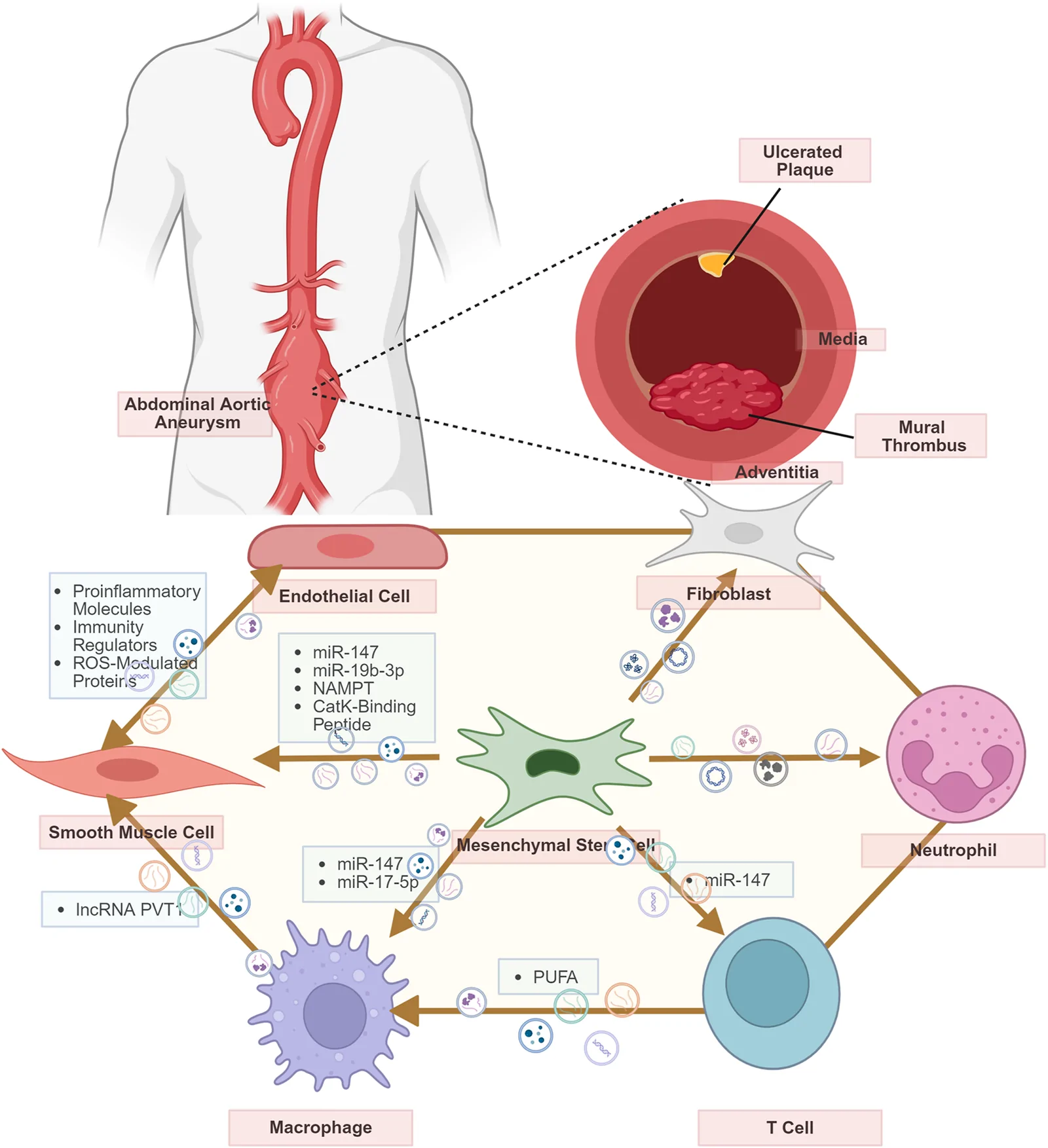
The biogenesis and uptake process of EVs. This schematic depicts the complex cellular landscape of AAA, comprising endothelial cells, VSMCs, fibroblasts, immune cells (macrophages, neutrophils, T cells), and MSCs. The diagram highlights the role of EVs as key mediators of intercellular communication by transferring specific bioactive molecules between these cells, which can either promote pathogenesis or deliver therapeutic cargo. Key structural features such as the intima, media, adventitia, and mural thrombus are also illustrated. CatK, Cathepsin K; miR, microRNA; NAMPT, Nicotinamide Phosphoribosyltransferase; PUFA, Polyunsaturated Fatty Acid; PVT1, Plasmacytoma Variant Translocation 1; ROS, Reactive Oxygen Species.

The abdominal aorta is a sophisticated, multilayered conduit whose integrity is essential for systemic circulatory function. Its wall is composed of three concentric layers: the tunica intima, media, and adventitia. The pathogenesis of AAA involves the progressive and localized dilation of this structure, a process frequently accompanied by the formation of an intraluminal thrombus (ILT) and significant inflammatory infiltration. The cellular landscape within the aneurysmal segment becomes markedly altered, comprising not only resident vascular cells—endothelial cells (ECs), VSMCs, and fibroblasts—but also a substantial influx of immune cells such as macrophages, neutrophils, and lymphocytes [38]. The physiological homeostasis of the aortic wall relies on exquisitely balanced communication and functional coordination among these diverse cell types (Figure 2).

The progression of AAA is fundamentally driven by the breakdown of this cellular harmony. A self-perpetuating cycle ensues, characterized by sustained inflammation, the phenotypic switching and eventual loss of contractile VSMCs, and the excessive degradation of the ECM by proteolytic enzymes [39–42]. Within this pathological microenvironment, EVs have been recognized as critical facilitators of intercellular communication. They act as bioactive cargo shuttles, transferring proteins, lipids, and regulatory nucleic acids between cells, thereby actively participating in the dysregulation of tissue homeostasis. A key emerging concept is the existence of a bidirectional regulatory loop between EVs and the ECM. Signals derived from the remodeled ECM can modulate EV biogenesis and molecular sorting, while EVs reciprocally deliver effector molecules that further degrade or reorganize the ECM [43, 44]. Accordingly, extracellular vesicles represent promising therapeutic targets for abdominal aortic aneurysm, given that their bioactive cargo is closely implicated in disease pathological mechanisms, and their phospholipid bilayer membrane structure confers high stability to encapsulated functional molecules.

## Application prospects of EVs in the treatment of AAA

### Main treatment strategies for AAA

AAA lacks disease-modifying pharmacotherapy: in real-world practice, definitive management still relies on open repair or EVAR once aneurysms reach threshold size or become symptomatic, whereas patients with small AAAs are largely managed by risk-factor control and imaging surveillance, despite the persistent risk of expansion and rupture and the substantial clinical, economic, and psychological burden of long-term follow-up [4, 45, 46]. This therapeutic gap reflects the systems-level nature of AAA progression, which is driven by intertwined processes—chronic inflammation and immune-cell infiltration, ECM degradation, and VSMC dysfunction/senescence—spanning multiple vascular and immune cell types [47–49]. Consequently, approaches aimed at a single molecular target have repeatedly shown limited translation, motivating interest in modalities capable of coordinately modulating pathogenic cellular networks. EVs offer a mechanistically plausible platform in this setting because they are membrane-enclosed carriers of bioactive cargo (RNAs, proteins, lipids) that can reprogram recipient-cell phenotypes and can be further engineered to enrich therapeutic payloads or enhance tissue targeting—features that conceptually match AAA’s multi-pathway pathology and the need for minimally invasive, potentially repeatable interventions [50, 51]. At the same time, any EV-based therapeutic rationale must be evaluated with translational realism, given known challenges such as rapid systemic clearance and the need for rigorous purification/functional attribution to ensure that observed effects are mediated by *bona fide* EVs rather than co-isolated non-vesicular components; these constraints are addressed in the subsequent sections on delivery, pharmacokinetics, and safety.

### The potential of EVs as drug delivery systems or therapeutic tools for AAA

EVs have emerged as promising drug delivery systems and cell-free therapeutic candidates in AAA models (Table 1). Their biocompatibility and relatively low immunogenicity make them attractive for vascular applications, as membrane encapsulation can protect labile cargos and support intercellular transfer of nucleic acids and proteins to lesion-relevant cells within the vascular wall. Across the current AAA therapeutic literature, EV preparations are most commonly obtained using differential centrifugation/ultracentrifugation-based workflows. Therapeutic efficacy is typically evaluated in established murine AAA models with lesion-level readouts in the abdominal aorta. Mechanistic validation is frequently performed in parallel using relevant vascular and immune cell systems—most prominently macrophages and VSMCs—and, in some studies, *ex vivo* vascular tissue preparations, enabling a closer linkage between EV exposure, pathway modulation, and phenotypic benefit [59].

**TABLE 1:** Therapeutic effects of EVs in preclinical AAA models.

Functional component	Donor cell	Level of evidence	Controls used	Therapeutic functions	Associated mechanism	References
miR-147 + EVs synergistic effect	hUC-MSCs	*In vivo*, *in vitro*	Cargo-molecule inhibitor/mimic	Attenuates AAA formation and vascular inflammation; preserves aortic structural integrity	Primarily via EV-delivered miR-147 to suppress macrophage activation; independently inhibits IL-17 secretion from CD4^+^ T cells	[52]
miR-17-5p	ADSCs, isolated from inguinal subcutaneous adipose tissue of C57BL/6 mice	*In vivo*, *in vitro*	Cargo-molecule inhibitor/mimic	Inhibits AAA formation; reduces pro-inflammatory cytokine release and macrophage pyroptosis; preserves elastic fibers	EV-delivered miR-17-5p targets TXNIP, suppressing the NLRP3 inflammasome and subsequent macrophage pyroptosis	[53]
Vesicle-mediated delivery	MSCs	*In vivo*, *in vitro*	No EV control	Suppresses AAA formation; reduces NETs release and SMCs ferroptosis	EVs redirect neutrophil NETosis to apoptosis, thereby inhibiting NET-mediated PI3K/AKT pathway suppression and SMC ferroptosis	[12]
Vesicle-associated biological effect	Rat pancreatic pathfinder cells	*In vitro*	Young/aged EVs	Enhances repair of mechanical/genotoxic injury in young/middle-aged cells; exerts senotherapeutic effects in aged cells	Promotes cell proliferation/migration; induces clearance of senescent cells followed by compensatory proliferation to restore homeostasis	[37]
CKBP surface targeting + EV platform	HBM-MSCs	*In vivo*, *in vitro*	Disrupted EVs/unmodified EVs/blank control	Enhances targeted binding to CatK-overexpressing aneurysmal cells; promotes vascular elastic matrix repair	Surface-conjugated CKBP improves targeting; EVs themselves regulate MMP2 and LOX to maintain matrix homeostasis	[36]
NAMPT	hiPSC-MSCs	*In vivo*, *in vitro*	Control EVs, NAMPT inhibitor/mimic, PBS blank control	Attenuates AAA formation; inhibits VSMC senescence and DNA damage; restores oxidative phosphorylation and ATP synthesis	EV-delivered NAMPT enhances NAD^+^ biosynthesis, activates SIRT1, and improves mitochondrial function to suppress VSMC phenotypic switch	[54]
Internal cargo and engineered EV-enhanced targeting	HBM-MSCs	*In vivo*, *in vitro*	With vs. without engineering modification	Enhances delivery of MSCEs to AAA; promotes elastic matrix regenerative repair, reduces oxidative stress, activates PI3K/Akt signaling; associated with aneurysm shrinkage/reversal	Magnetic field navigation enriches at aneurysm wall; catalase-driven chemotaxis toward H_2_O_2_-rich AAA microenvironment increases uptake; downstream effects include reduced ROS and PI3K/Akt pathway activation supporting matrix repair and anti-apoptotic signaling	[11]
Internal cargo	MSCs	*In vivo*, *in vitro*	No EV control	Impedes AAA development in AngII-induced Apoe^−/−^ mice and CaCl_2_-induced C57BL/6 mice; reduces maximal abdominal aortic diameter and elastic fiber degradation; promotes macrophage M2 polarization	MSC-Exo suppress CD74 in macrophages, decreasing PKM2 and modulating the TSC2–mTOR–AKT pathway to drive macrophage polarization toward M2 under inflammatory conditions	[55]
miR-221-5p	IL-4–polarized BMDMs	*In vivo*, *in vitro*	Free cargo, cargo-depleted exosomes	Reduces AAA incidence and maximal aortic diameter; improves elastic fiber continuity; increases α-SMA; decreases macrophage infiltration; alleviates inflammatory responses	EV-delivered miR-221-5p targets PARP-1 mRNA to suppress PARP-1, releasing PP-1α and inhibiting JNK/c-Jun signaling, thereby shifting macrophage polarization toward M2 and restraining AAA progression	[56]
Internal cargo and silk–iron packaged extracellular vesicles	ASCs	*In vivo*, *in vitro*	With vs. without engineering modification	Enables magnetic localization and controlled release/uptake of ASC-EVs	Biomaterial-enabled magnetic localization and depot-like release of encapsulated EVs	[57]
OPN targeting + miR-149-5p	hPMSCs	*In vivo*, *in vitro*	Cargo-molecule inhibitor/mimic	Reduces AAA formation in both AngII- and elastase-induced murine models; suppresses VSMC senescence	Targeted homing/retention at aneurysmal sites via OPN-binding; EV miR-149-5p downregulates Nat10, inhibiting VSMC senescence (miR-149-5p/Nat10/senescence axis)	[58]

hUC-MSCs, Human Umbilical Cord-derived Mesenchymal Stem Cells; AoSMCs, Aortic Smooth Muscle Cells; ADSCs, Adipose-Derived Stem Cells; TXNIP, Thioredoxin-Interacting Protein; NLRP3, NLR Family Pyrin Domain Containing 3; NETs, Neutrophil Extracellular Traps; SMCs, Smooth Muscle Cells; HASMCs, Human Aortic Smooth Muscle Cells; HBM-MSCs, Human Bone Marrow-derived Mesenchymal Stem Cells; CatK, Cathepsin K; CKBP, Cathepsin K-Binding Peptide; MMP2, Matrix Metalloproteinase 2; LOX, Lysyl Oxidase; PI3K: Phosphatidylinositol 3-kinase; Akt: Protein kinase B; ADMSCs, Adipose-Derived Mesenchymal Stem Cells; MST4, Mammalian Sterile-20-like kinase 4; ERK, Extracellular Signal-Regulated Kinase; Drp1, Dynamin-related protein 1; ROS, Reactive Oxygen Species; hiPSC-MSCs, Human Induced Pluripotent Stem Cell-derived Mesenchymal Stem Cells; NAMPT, Nicotinamide Phosphoribosyltransferase; NAD+, Nicotinamide Adenine Dinucleotide; SIRT1, Sirtuin 1; ATP, Adenosine Triphosphate; MSCEs, MSC exosomes; CD74: Cluster of Differentiation 74; PKM2: Pyruvate Kinase M2; TSC2: Tuberous Sclerosis Complex 2; mTOR: Mechanistic Target of Rapamycin; IL-4, Interleukin-4; PARP-1: Poly (ADP-Ribose) Polymerase 1; PP-1α: Protein Phosphatase 1α; JNK: c-Jun N-terminal Kinase; c-Jun: cellular Jun; OPN: Osteopontin; Nat10: N-Acetyltransferase 10.

A macrophage-centered immunomodulatory theme is supported by multiple independent studies. Spinosa M et al. reported that MSCs and MSC-derived EVs exhibit comparable protective effects in AAA and proposed that endogenous miR-147 signaling is insufficient during disease progression. Interestingly, the authors also identified a miR-147-independent therapeutic potential of MSC-EVs, as these vesicles suppressed IL-17 secretion by CD4^+^ T cells and reduced neutrophil and T-cell infiltration. Overall, MSC-EV delivery was associated with reduced AAA formation and vascular inflammation, as well as preservation of aortic structural integrity. Validation included murine abdominal aortic lesions and complementary assays in human aortic explants and *in vitro* recipient systems, including AoSMCs, CD11b^+^ macrophages, and CD4^+^ T cells. The proposed mechanism emphasized EV-delivered miR-147-mediated suppression of macrophage activation, with an additional effect on IL-17 secretion from CD4^+^ T cells [52]. Besides, Hu J et al. reported that adipose-derived MSC-EVs alleviated aortic dilation, improved elastic fiber integrity, and increased survival in an Ang II–induced ApoE^−/−^ mouse AAA model. Mechanistic studies indicated macrophage uptake of EVs and delivery of miR-17-5p, which targets TXNIP and suppresses NLRP3 inflammasome activation, thereby reducing inflammatory cytokine release and macrophage pyroptosis; importantly, inhibitor- and mimic-based validation further supported attribution of this effect to EV cargo [53].

Evidence for macrophage polarization control has also become more explicit, which has recently been reinforced by a study that tested MSC-derived exosomes across two AAA models. Xu et al. evaluated MSC-Exo in Ang II–induced AAA in Apoe^−/−^mice and in CaCl2-induced AAA in C57BL/6 mice, reporting reduced AAA development alongside macrophage phenotype changes consistent with anti-inflammatory polarization. Mechanistic analyses supported CD74 suppression as an upstream node linked to PKM2 and the TSC2–mTOR–AKT pathway, strengthening the plausibility of a CD74-centered macrophage reprogramming mechanism in AAA [55]. A complementary approach is to use immune-cell-derived EVs as the therapeutic product. Ma et al. reported that M2 macrophage-derived EVs reduced AAA incidence and maximal aortic diameter in Ang II–infused ApoE^−/−^ mice, improved fiber continuity, increased α-SMA, and reduced macrophage infiltration in aneurysmal lesions. RNA sequencing highlighted miR221-5p enrichment in M2-EVs, and miR221-5p antisense oligonucleotides attenuated protection *in vivo*. Mechanistic experiments suggested that miR221-5p targets PARP-1 and modulates macrophage polarization through a PARP-1/PP-1α/JNK/c-Jun axis, nominating another tractable EV–miRNA–signaling module for inflammatory control in AAA [60].

Beyond macrophage-centered mechanisms, Chen L et al. reported that MSC-EVs reduced AAA formation in an Ang II–induced ApoE^−/−^ model and proposed a pathway linking neutrophil behavior to smooth muscle cell injury. In this framework, EVs were suggested to redirect neutrophil NETosis toward apoptosis, thereby reducing NET-associated suppression of PI3K/AKT signaling and limiting VSMC ferroptosis-related damage. Mechanistic validation emphasized neutrophils (NET formation) and smooth muscle cell systems to connect immune modulation with vascular protection [12]. In addition, studies have also been conducted on the relationship between aging and AAA. Panagiotou N et al. investigated EVs from rat pancreatic Pathfinder cells and observed context-dependent effects *in vitro* using human dermal fibroblast–MSC coculture systems, including enhanced repair responses in younger/middle-aged cells and senotherapeutic-like effects in aged cells [37]. Although not a direct AAA *in vivo* therapeutic demonstration, these findings support the broader concept that EVs can modulate tissue repair and senescence programs that are relevant to vascular aging biology.

Taken together, current preclinical evidence—predominantly using MSC-derived EVs—supports the concept that EV-based interventions can attenuate AAA development through convergent effects on macrophage activation and polarization programs, VSMC senescence/mitochondrial stress pathways, and immune–vascular cross-talk such as NET-associated injury. These findings provide a rationale for refining EV products and delivery strategies to better match AAA’s focal, multi-cellular pathology.

### Advances in the use of engineered EVs for the treatment of AAA

Engineered EVs are a novel therapeutic delivery platform created through the targeted modification of natural EVs using biological or chemical methods [61, 62]. By modifying the membrane surface for targeted delivery or loading of therapeutic cargo, their therapeutic efficacy and ability to home to disease sites can be significantly enhanced, providing a powerful technological platform for achieving precision medicine.

As an example of cargo engineering, Ouyang Y et al. built on the observation that NAMPT can restore NAD^+^ biosynthesis, improve mitochondrial oxidative phosphorylation, and delay VSMC senescence and pathological phenotypic switching—processes closely linked to AAA progression. Methodologically, the authors used gene editing to overexpress NAMPT in MSCs and then harvested NAMPT-enriched EVs (NAMPT-EVs), with EV isolation performed by differential centrifugation. The evaluation strategy was also designed to connect lesion and cell level readouts: *in vivo* efficacy was assessed in the abdominal aorta of an Ang II induced ApoE^−/−^ mouse AAA model. At the same time, *in vitro* mechanistic validation focused on HVSMCs from healthy donors and AAA patients as recipient cells. Using this engineered product, the study showed that, compared with free NAMPT protein and control EVs, NAMPT-EVs enabled more efficient functional delivery of NAMPT, which is otherwise prone to degradation and difficult to internalize, thereby supporting the concept that EV packaging enhances functional NAMPT delivery and improves the translational relevance of this therapeutic strategy [54].

In parallel, surface functionalization addresses another major bottleneck: after systemic administration, EVs often show limited lesion accumulation due to poor targeting and rapid clearance. Sajeesh S et al. developed a targeting-enhanced MSC-EV platform by coupling cathepsin K-binding peptides (CKBP) onto the EV surface using click chemistry [63]. In this work, the MSC-EVs intended for surface conjugation were first prepared using ultracentrifugation combined with differential centrifugation, and the targeting performance was tested in recipient systems that model aneurysm-associated SMC injury and matrix damage—namely elastase-induced rat abdominal aortic aneurysm SMCs *in vitro* and elastase-injured porcine carotid arteries *ex vivo*. With this design, the authors reported improved binding to CatK-overexpressing aneurysmal cells and functional effects consistent with aneurysm-wall stabilization, including inhibition of MMP2 activity and enhancement of LOX activity, thereby linking lesion homing with reduced elastolysis and promoted elastic fiber regeneration; comparisons with disrupted EVs, unmodified EVs, and blank controls further supported these conclusions.

Recent work has further strengthened the concept that AAA-focused engineering should explicitly solve delivery and retention, rather than only increasing cargo potency. Jia X et al. started from the pathobiological observation that VSMC senescence is a key driver of aneurysm remodeling and that unmodified EVs are often cleared rapidly with insufficient lesion exposure [58]. They isolated hPMSC-EVs via differential ultracentrifugation, then introduced an osteopontin (OPN)-targeted peptide onto EV membranes using a mild hydrophobic insertion strategy based on cholesterol-PEG conjugates. This design exploited the enrichment of OPN in AAA lesions and in senescent VSMCs, and recipient validation emphasized senescent VSMCs *in vitro* and aneurysmal aortic tissue *in vivo* (Ang II–and elastase-induced murine AAA models), with imaging evidence supporting lesion accumulation and colocalization with α-SMA^+^ VSMCs after intravenous dosing. Therapeutically, OPN-targeted hPMSC-EVs reduced AAA formation across both models while suppressing VSMC senescence, indicating that OPN-mediated lesion homing and miR-149-5p-dependent bioactivity act synergistically in this engineered EV platform. Mechanistic mapping connected the effect to downregulation of Nat10, mediated by EV-carried miR-149-5p, establishing a miR-149-5p/Nat10/senescence axis as an engineering-relevant pathway node for AAA.

Complementary delivery-centric engineering has been explored using hybrid “exosome–nanomotor” constructs designed for the high-flow arterial environment. Wang L et al. isolated BM-MSC exosomes by ultracentrifugation, then coupled them onto an asymmetric, magnetically responsive mesoporous silica module embedded with SPION, with additional loading of catalase to enable chemotactic behavior in H_2_O_2_-rich aneurysmal microenvironments [11]. The platform was evaluated in an elastase-induced rat AAA model using tail-vein administration with external magnetic field guidance, focusing on aneurysmal aorta targeting and VSMC uptake. This dual-drive strategy increased aortic accumulation while reducing nonspecific organ distribution, and was accompanied by functional readouts consistent with elastic matrix repair and aneurysm regression, including reduced post-treatment aneurysm diameter relative to pretreatment, improved histological features, and pathway signals consistent with PI3K/Akt activation. The SPION module also enabled MRI contrast readouts, supporting a theranostic-oriented delivery concept for AAA.

Other direction integrates EVs with depot-like biomaterials to support localization and controlled release. Marini AX et al. developed silk–iron packaged extracellular vesicles (SIPEs) that combine regenerated silk fibroin with iron oxide nanoparticles to permit magnetic localization, while encapsulating ASC-derived EVs [57]. The authors demonstrated magnetic localization of SIPEs *in vitro* and localization of related silk–iron microparticles (SIMPs) in an *in vivo* rat AAA setting, with evidence of particle presence around the aorta using Prussian blue staining. Release and uptake feasibility was supported by detection of EV-associated CD63^+^ events and uptake assays; however, the biological activity of releasates was diminished relative to unencapsulated controls, and the work explicitly pointed to loading efficiency and formulation optimization as the main limiting step. This study frames an important practical trade-off for AAA: improved spatial control may come at the cost of reduced effective payload unless encapsulation and release kinetics are optimized.

Taken together, current AAA studies suggest that engineered EVs can potentially improve therapeutic efficacy through enhanced payload design (e.g., NAMPT-enriched EVs, miRNA-mediated anti-senescence programs) and/or improved lesion exposure (peptide-guided targeting, magnetic guidance, chemotaxis-enabled secondary targeting, magnetically localizable depots), with recipient systems increasingly emphasizing VSMCs and aneurysmal aortic tissue as the key target compartment (Table 1).

**FIGURE 3:**
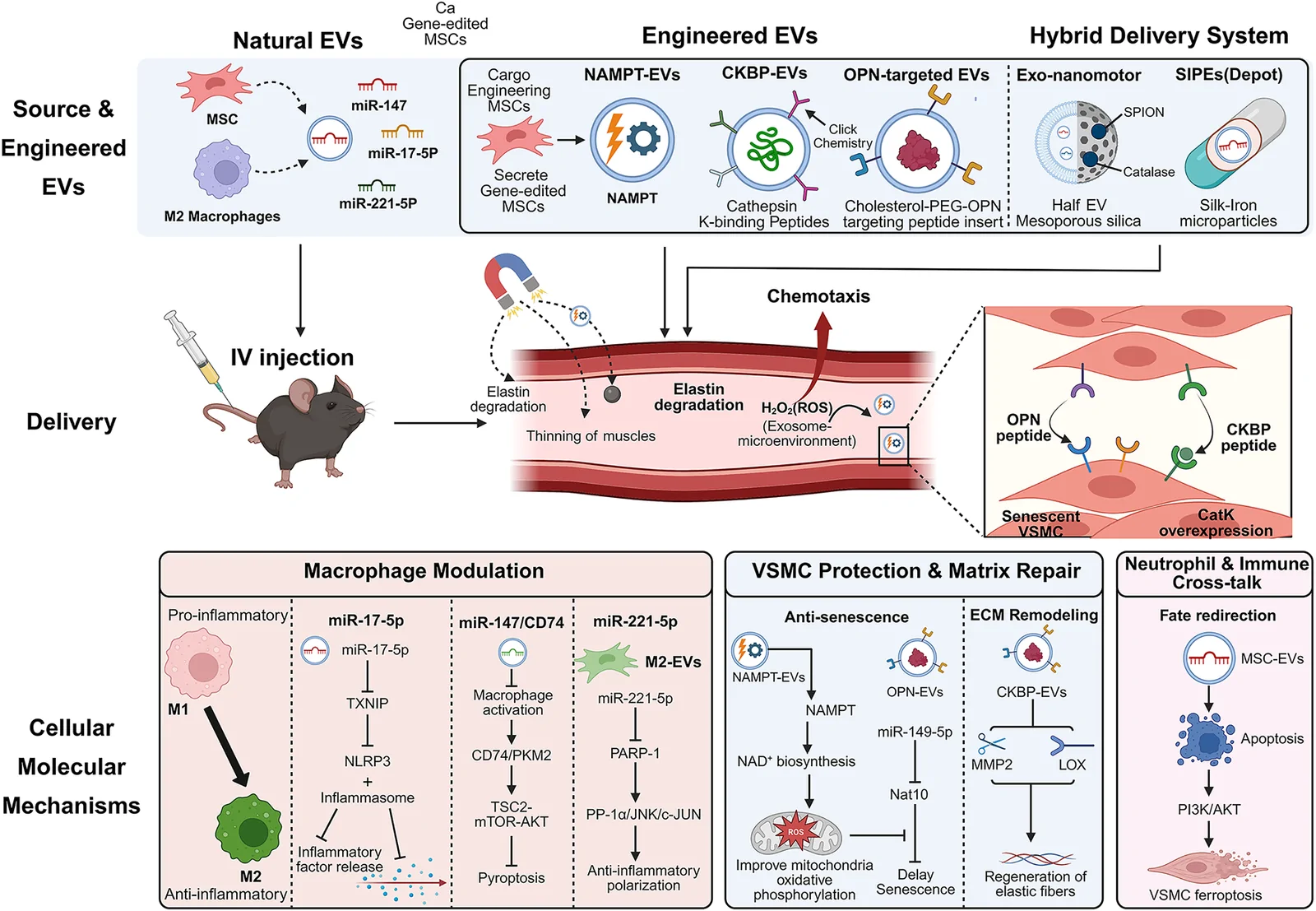
Therapeutic potential and engineering strategies of EVs in AAA. The diagram summarizes the major EV-based therapeutic approaches for abdominal aortic aneurysm (AAA), including natural EVs derived from mesenchymal stromal/stem cells (MSCs) or immune cells, as well as engineered EV platforms designed to enhance cargo delivery and lesion targeting. Therapeutic EVs can attenuate AAA progression by suppressing macrophage-driven inflammation and pyroptosis, modulating macrophage polarization, inhibiting neutrophil extracellular trap (NET)-associated vascular injury, protecting vascular smooth muscle cells (VSMCs) from senescence, ferroptosis, apoptosis, and mitochondrial dysfunction, and reducing extracellular matrix (ECM) degradation. Engineered EVs may further improve therapeutic efficacy through cargo enrichment, peptide-mediated targeting, magnetic guidance, chemotaxis-enabled delivery, and biomaterial-assisted retention, thereby enhancing aneurysm-wall accumulation and target engagement. Collectively, EV-based strategies provide a cell-free therapeutic framework for modulating the inflammatory–degenerative network of AAA and promoting vascular repair. AAA, Abdominal Aortic Aneurysm; ECM, Extracellular Matrix; EVs, Extracellular Vesicles; MSCs, Mesenchymal Stromal/Stem Cells; NET, Neutrophil Extracellular Trap; VSMCs, Vascular Smooth Muscle Cells.

The therapeutic effects observed in AAA studies arise from multiple potential sources, including the vesicle as a delivery vehicle, membrane- or surface-associated properties, and specific cargo molecules such as miRNAs or proteins. This diversity of mechanisms broadens the traditional view of EVs as simple carriers and highlights their potential as biologically active therapeutic entities. However, the strength of evidence varies across studies, and mechanistic interpretation should be approached with caution in light of the controls used, such as free cargo, disrupted EVs, cargo-depleted EVs, or appropriate donor-cell and vehicle controls. Accordingly, the reported effects of EV-based interventions should be evaluated by integrating cargo attribution, vesicle-associated activity, and the rigor of mechanistic validation. In summary, accumulating preclinical studies support EVs—particularly MSC-derived and engineered EVs—as promising cell-free modalities to modulate the inflammatory–degenerative network of AAA, with convergent benefits on macrophage programs, VSMC integrity, and matrix remodeling (Figure 3). These advances provide a rationale for developing mechanism-informed EV products and delivery strategies aimed at slowing aneurysm growth and ultimately reducing rupture risk.

## Mechanistic basis for EV-targeted interventions in AA--atherogenic EV axes and druggable nodes

### The main pathological mechanisms of AAA

AAA pathogenesis is driven by intertwined inflammation, ECM degradation, and VSMC dysfunction. Beyond serving as therapeutic carriers, endogenous EVs represent an upstream layer of intercellular communication that can actively shape these pathological processes [64, 65]. In this section, we summarize basic mechanistic studies that were not covered in the therapy-focused sections, and highlight druggable EV axes that may inspire future intervention strategies.

### Mechanistic exploration of EVs in AAA

Although many studies to date have not focused on AAA therapy, they still provide important insights into how endogenous EVs shape the AAA microenvironment (Table 2). These findings collectively suggest that EV-mediated intercellular communication can link risk factors and immune activation to ECM degradation and VSMC injury, thereby forming a pathogenic signaling network in AAA lesions.

**TABLE 2:** Mechanistic research on the role of EVs in AAA pathogenesis.

EV components	Donor cell	Level of evidence	Key mechanisms	Application potential	References
ADAM10 and ADAM17	Neutrophils (HL-60 human promyelocytic leukemia cells differentiated into neutrophils)	Clinical, *in vitro*	Neutrophil-derived MVs carry active ADAM10/ADAM17, which mediate aortic wall degradation. Tobacco smoke induces the release of these MVs	Serve as novel biomarkers; inhibiting ADAM10/17 or their parent MVs could mitigate AAA progression, especially in smokers	[66]
miR-106a	Plasma, tissue	Clinical, *in vitro*	Exosomal miR-106a induces VSMC apoptosis and targets TIMP-2, upregulating MMP-2/MMP-12 to accelerate ECM degradation	Serves as a diagnostic biomarker; inhibiting exosomal miR-106a or its TIMP-2/MMPs axis offers a potential therapy	[67]
N/A	THP-1 cell-derived macrophages	Clinical, *in vivo*, *in vitro*	Macrophage-derived EVs promote MMP-2 expression in VSMCs via JNK/p38 pathways, accelerating ECM degradation. GW4869 inhibits EV generation	GW4869 is a candidate therapeutic; targeting macrophage EVs or the JNK/p38-MMP-2 axis offers a novel intervention strategy	[68]
PUFA-containing phospholipids	PKM2-activated T lymphocytes; T lymphocytes; lasma	Clinical, *in vivo*, *in vitro*	PKM2-activated T-cell-derived EVs, enriched in PUFA-phospholipids, are internalized by macrophages to promote iron accumulation and lipid peroxidation, aggravating AAA.	Targeting T-cell PKM2, EV biogenesis, or the macrophage iron-lipid peroxidation axis offers a novel therapeutic strategy; EVs or PKM2 serve as diagnostic biomarkers	[13]
Proinflammatory-related molecules, trained immunity regulators, ROS-modulated proteins	Pathological HASMCs, HMECs	Clinical, *in vivo*, *in vitro*	Pathological aortic cell-derived EVs deliver proinflammatory and trained immunity regulators to activate macrophages and VSMCs, sustaining chronic inflammation	Targeting EV biogenesis or its key components offers a therapeutic strategy; EV-derived molecules serve as diagnostic biomarkers	[69]
lncRNA PVT1	M1-polarized macrophages	*In vitro*	M1 macrophage-derived EVs deliver lncRNA PVT1, which sponges miR-186-5p to upregulate HMGB1, promoting VSMC inflammation and pyroptosis	Targeting exosomal lncRNA PVT1 or the miR-186-5p/HMGB1 axis provides a novel therapeutic strategy; lncRNA PVT1 serves as a diagnostic biomarker	[70]
FOSB protein	Cells expressing FOSB	*In vivo*, *in vitro*	FOSB-containing EVs promote VSMC phenotypic switch, upregulate MMP2/MMP9, and correlate with immune cell infiltration to drive AAA progression	FOSB is a promising diagnostic biomarker; targeting FOSB or its mediated pathways may mitigate AAA by inhibiting VSMC dysfunction and ECM degradation	[56]

EVs, Extracellular Vesicles; MVs, Microvesicles; AAA, Abdominal Aortic Aneurysm; miR, microRNA; VSMC, Vascular Smooth Muscle Cell; TIMP-2, Tissue Inhibitor of Metalloproteinase 2; MMP, Matrix Metalloproteinase; ECM, Extracellular Matrix; THP-1, Human Acute Monocytic Leukemia Cell Line; PKM2, Pyruvate Kinase M2; PUFA, Polyunsaturated Fatty Acid; HASMCs, Human Aortic Smooth Muscle Cells; HMECs, Human Microvascular Endothelial Cells; ROS, Reactive Oxygen Species; lncRNA: long non-coding RNA; PVT1: Plasmacytoma Variant Translocation 1; HMGB1, High Mobility Group Box 1; FOSB, FosB Proto-Oncogene, AP-1 Transcription Factor Subunit.

Folkesson M et al. analyzed EVs isolated by ultracentrifugation combined with differential centrifugation and showed that EVs present in AAA contexts are largely linked to neutrophil activity [66]. Using neutrophil-derived vesicles from HL-60–differentiated neutrophils, they identified ADAM10/17-containing microvesicles and observed their presence in the abluminal layer of the intraluminal thrombus (ILT) and the aortic wall of human AAA. Mechanistically, tobacco smoke extract promoted the release of these protease-bearing vesicles, which were proposed to accelerate ECM degradation and weaken the aneurysmal wall. This “smoking–neutrophil EV–ADAM protease” axis nominates EV-associated ADAM activity as a tractable intervention point, particularly in smoking-related AAA.

EV cargo–driven injury signaling has also been demonstrated at the level of ncRNAs acting directly on VSMCs. Han Z et al. examined plasma- and tissue-derived EVs prepared using ultracentrifugation and differential centrifugation, and linked exosomal miR-106a to AAA-associated remodeling [67]. Recipient systems included the abdominal aortic wall of patients *in vivo* and human VSMCs *in vitro*, enabling a lesion-to-cell mechanistic connection. In HVSMCs, miR-106a targeted TIMP-2, increased MMP-2/MMP-12 secretion, and promoted apoptosis, providing a coherent route from EV cargo to protease imbalance and ECM degradation. This axis supports both biomarker relevance and the therapeutic logic of inhibiting exosomal miR-106a or its downstream TIMP-2/MMP pathway.

Macrophage-to-VSMC EV communication provides another direct route to protease induction. Wang Y et al. isolated macrophage-derived EVs using ultracentrifugation and differential centrifugation from THP-1 cell–derived macrophages, then evaluated their effects across the abdominal aorta of CaPO_4_-induced AAA in C57BL/6 mice *in vivo* and HVSMCs *in vitro* as recipient systems [68]. They reported that macrophage EVs activated JNK/p38 signaling and increased MMP-2 expression in VSMCs, supporting an immune-to-vascular axis that accelerates ECM degradation. Notably, they also highlighted pharmacologic modulation of vesicle production: the exosome-generation inhibitor GW4869 attenuated AAA-related signaling in this context, emphasizing EV biogenesis as a druggable upstream node.

Beyond protein and RNA cargo, EV lipid composition can amplify inflammatory injury through metabolic stress. Dang G et al. used EVs isolated by ultracentrifugation and differential centrifugation and showed that EVs derived from PKM2-activated T lymphocytes are enriched in PUFA-containing phospholipids [13]. Recipient systems included the abdominal aortic wall of elastase-induced AAA in C57BL/6J mice *in vivo* and murine macrophages as well as human THP-1 monocytes/macrophages *in vitro*. In macrophages, these EVs promoted iron accumulation and lipid peroxidation, increasing migration and inflammatory amplification. The observation that GW4869 alleviated AAA progression in their model further supports the feasibility of targeting EV biogenesis/release or the downstream macrophage iron–lipid peroxidation axis.

A broader systems-level perspective comes from studies examining EV programs released by pathological aortic cells. Lu Y et al. isolated EVs using ultracentrifugation and differential centrifugation from pathological human aortic smooth muscle cells (HASMCs) and HMECs and profiled proinflammatory-related molecules, trained-immunity regulators, and ROS-modulated proteins [69]. Recipient systems spanned the abdominal aorta of Ang II–and elastase-induced AAA mice *in vivo*, and murine VSMCs/macrophages and human HMECs *in vitro*. These data support a model in which aortic cell–derived EVs sustain chronic inflammation by activating macrophages and VSMCs, while also nominating EV biogenesis or key cargo components as potential intervention nodes.

EV-mediated macrophage programming can also be transmitted to VSMCs through lncRNA cargo. Zhang J et al. prepared EVs via ultracentrifugation and differential centrifugation from M1-polarized macrophages and tested their effects in HASMCs *in vitro* as recipient cells [70]. They reported that exosomal lncRNA PVT1 sponges miR-186-5p and upregulates HMGB1, promoting inflammatory activation and pyroptosis-like injury in VSMCs. This provides a tractable EV–lncRNA–miRNA–effector pathway that can be targeted at the cargo level or at the HMGB1 axis.

Finally, EV-associated transcriptional regulators may contribute to VSMC phenotypic switching and protease induction. Ma X et al. isolated EVs by ultracentrifugation and differential centrifugation from cells expressing FOSB, and evaluated their association with disease in recipient systems that included the abdominal aortic wall of Ang II–induced AAA mice *in vivo* and primary VSMCs *in vitro* [56]. FOSB-containing EVs were linked to enhanced VSMC phenotypic switching, increased MMP2/MMP9, and correlation with immune infiltration, collectively supporting a role for EV-borne transcriptional programs in driving ECM degradation and wall remodeling.

**FIGURE 4:**
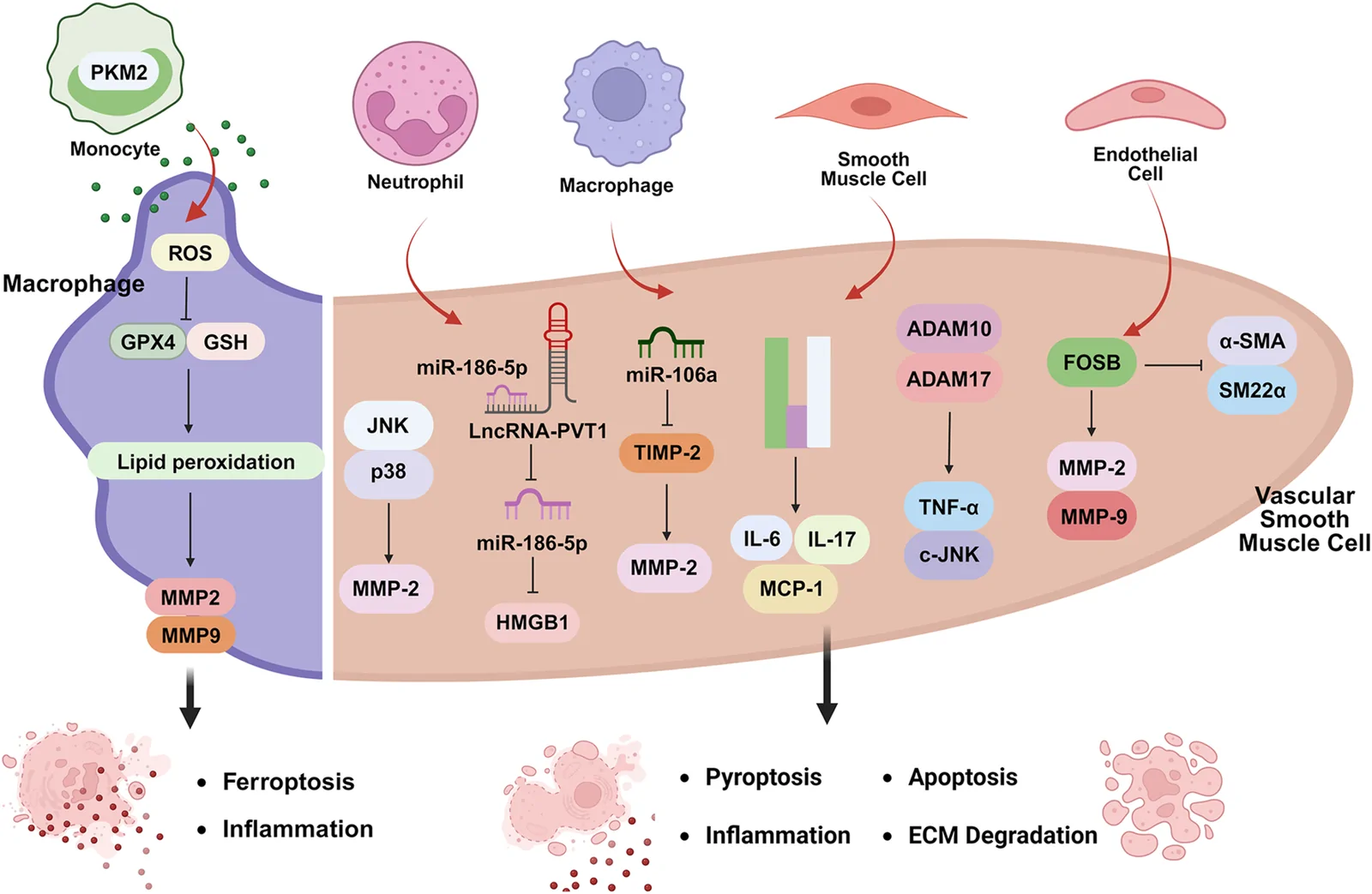
Mechanisms of EV-mediated intercellular communication in AAA pathogenesis. This schematic illustrates the complex roles of EVs in driving AAA progression through key pathological processes. EVs derived from various cells (e.g., T cells, macrophages, neutrophils) transfer specific cargoes to recipient cells including VSMCs, macrophages, and endothelial cells. These interactions activate signaling pathways, regulate gene expression, and modulate enzyme activities, collectively promoting critical events in AAA development: inflammation, pyroptosis, ferroptosis, apoptosis, and ECM degradation. Key molecules involved are indicated. ADAM, A Disintegrin And Metalloproteinase; ECM, Extracellular Matrix; GPX4, Glutathione Peroxidase 4; GSH, Glutathione; HMGB1, High Mobility Group Box 1; IL, Interleukin; JNK, c-Jun N-terminal Kinase; MCP-1, Monocyte Chemoattractant Protein-1; miR, microRNA; MMP, Matrix Metalloproteinase; PKM2, Pyruvate Kinase M2; PUFA, Polyunsaturated Fatty Acid; ROS, Reactive Oxygen Species; SM22α, Smooth Muscle Protein 22-alpha; TIMP-2, Tissue Inhibitor of Metalloproteinase-2; TNF-α, Tumor Necrosis Factor-alpha; α-SMA, Alpha-Smooth Muscle Actin.

Collectively, these mechanistic studies support a model in which EVs from immune and vascular-associated sources coordinate inflammatory amplification, protease-driven ECM breakdown, and VSMC dysfunction in AAA, while also highlighting intervention opportunities at the levels of EV biogenesis/release, EV uptake into lesion-relevant recipient cells, and specific pathogenic cargos/pathways (Figure 4).

## Discussion

### Current challenges associated with the clinical application of EVs in AAA

#### Evidence attribution and product integrity as prerequisites for translation

Before EV-based biomarkers or therapeutics can be credibly translated into AAA clinical practice, a fundamental prerequisite is robust attribution—i.e., demonstrating that diagnostic signals or biological effects arise from *bona fide* EVs rather than co-isolated non-vesicular components. In the broader EV field, co-purification of non-vesicular nanoparticles (NVNs) such as lipoproteins and protein/RNA complexes is a well-recognized confounder, particularly for commonly used ultracentrifugation-based workflows [71]. Without fit-for-purpose controls to assess vesicle integrity and exclude major contaminants, both mechanistic interpretation and product reproducibility can be undermined.

For AAA, this attribution requirement applies across the entire translational spectrum. In biomarker studies, inadequate exclusion of NVNs may inflate or distort candidate signatures. In therapeutic studies, insufficient purity and integrity assessment can complicate causal attribution of observed benefits and weaken the interpretability of dose–response relationships [71]. Therefore, AAA-EV investigations should increasingly adopt attribution-aware characterization strategies that match the intended use, including contaminant assessment, vesicle integrity controls, and orthogonal verification approaches, alongside transparent reporting consistent with community guidance. These attribution and integrity issues directly influence biomarker reliability, contributing to an evidence base that is currently not yet decision-grade for AAA clinical use.

#### Unresolved origins, cargo composition, and *in vivo* dynamics in AAA

AAA lesions involve multiple vascular and immune cell types, and EVs released under pathological conditions likely reflect diverse tissue origins and state-dependent cargo programs. However, systematic mapping of tissue/cell-of-origin, cargo evolution across disease stages, and compartment-specific dynamics remains limited. These gaps constrain interpretability for both biomarkers and therapeutics.

Moreover, *in vivo* dynamics—including biodistribution, half-life, and clearance mechanisms—remain a central translation question. Systemically administered EVs can exhibit rapid clearance and prominent uptake by clearance organs, raising practical questions about target-site exposure at aneurysmal tissue and the durability of downstream effects [72]. For AAA, where pathology is anatomically focal, translation will benefit from study designs that explicitly quantify tissue-level exposure and incorporate compartment-resolved readouts that link EV delivery to lesion biology.

#### Delivery feasibility, target engagement, and translational study design for EV therapeutics

A recurring translational bottleneck is that lesion-level delivery and mechanistic engagement are often inferred rather than demonstrated. Proposed therapeutic mechanisms—such as macrophage reprogramming, suppression of NET-associated injury, mitigation of VSMC senescence, or modulation of protease-driven ECM degradation—implicitly assume EV uptake by specific recipient cells within AAA lesions. However, direct *in vivo* evidence of cell-specific uptake and compartment-resolved engagement remains limited in many studies [73].

To strengthen translational credibility, future AAA-EV therapeutic studies should predefine the target compartment, intended recipient cell populations, and engagement biomarkers measurable within lesion tissue, so as to establish a clearer linkage between delivery, mechanism, and phenotype. Reporting of key translational parameters should also be standardized, including animal model choice, group sizes, dose metrics, dosing frequency/duration, route of administration, and clinically meaningful endpoints. These design features will be essential for cross-study comparison and for rational optimization of dosing regimens and delivery strategies.

#### Translation-enabling infrastructure: Standardization, quality/safety, and regulatory–manufacturing readiness

Clinical translation of EV-based diagnostics and therapeutics in AAA depends not only on biological efficacy, but also on reliable standardization, quality control, safety assessment, and scalable manufacturing. At present, EV isolation and characterization methods remain highly variable, including ultracentrifugation, size-exclusion chromatography, density gradients, precipitation, immunoaffinity capture, and microfluidic approaches. These methods may enrich different EV subpopulations and produce differences in purity, yield, and cargo profiles, making results difficult to compare across laboratories [74]. Therefore, AAA-related EV studies should follow community guidelines such as MISEV2023 and clearly report sample source, isolation method, storage condition, characterization strategy, and normalization approach [29]. Cross-cohort validation and platform harmonization will be essential before EV biomarkers can become clinically reliable tools.

Pre-analytical and storage conditions also require careful control. EV recovery and cargo measurement can be affected by buffer composition, storage duration, freeze–thaw cycles, and sample handling procedures. For example, simple PBS storage may reduce EV recovery, whereas optimized formulations containing protective additives may improve EV preservation [75]. However, additives such as albumin can also influence downstream proteomic analysis and should therefore be reported and interpreted carefully. For both diagnostic and therapeutic studies, detailed documentation of handling conditions is necessary to improve reproducibility.

Quality and safety are equally important for EV-based therapeutics. EVs should not be regarded as automatically safe, because their cargo can be influenced by donor-cell type, culture conditions, cellular stress, and engineering procedures. In AAA, where treatment may need to be repeated or long-term, preclinical studies should evaluate immune activation, coagulation effects, liver and spleen accumulation, and organ toxicity [76]. In addition, EV products require more than marker-based identification. They need fit-for-purpose quality control, including identity, purity, sterility, contaminant assessment, and potency assays linked to AAA mechanisms, such as inhibition of macrophage inflammation, reduction of protease activity, or protection against VSMC senescence and oxidative stress.

Finally, regulatory and manufacturing issues will strongly influence clinical application. Depending on their intended use and engineering level, EV products may be classified as biological products, drug delivery systems, or diagnostic devices. However, regulatory pathways for engineered EVs are still not fully harmonized [77]. Scalable production also remains challenging, because conventional two-dimensional cell culture often provides limited yield and batch consistency, while bioreactor-based systems still require further optimization for clinical-grade production. Ethical issues, including biospecimen consent, data privacy, and psychological impact of predictive AAA biomarker disclosure, should also be considered [78]. Overall, EV-based AAA applications will require not only stronger mechanistic evidence, but also standardized workflows, robust quality systems, scalable manufacturing, and clear regulatory strategies.

#### Evidence maturity and clinical validity gaps: from discovery signals to decision-grade tools

Compared with EV-based therapeutics, EV biomarkers for AAA diagnosis and monitoring may be closer to clinical application because they rely on relatively accessible biofluid samples and can be linked more directly to clinical readouts (Table 3). Specifically, most reported candidates have been identified from plasma or serum derived EVs and include both proteins and noncoding RNAs, such as ferritin light chain, ficolin-3, miR-106a, miR-122-5p, and miR-483-5p [67, 79–83]. These studies collectively suggest that EV-associated molecules may help distinguish AAA from non-AAA populations and may also capture biological processes relevant to extracellular matrix remodeling, vascular smooth muscle cell injury, inflammation, and complement activation. However, most currently available studies remain cross-sectional and diagnostic in orientation, with relatively few directly addressing clinically actionable outcomes such as aneurysm growth, rupture risk, or post-intervention complications. An exception is the use of activated endothelial-derived EVs for post-EVAR endoleak monitoring, which begins to move EV biomarkers toward longitudinal and management-relevant applications [84]. However, most evidence remains at the discovery stage, and no EV-based biomarker has yet become a decision-grade clinical tool for AAA management [85].

**TABLE 3:** Representative EV-based biomarker studies in AAA and clinical relevance.

Sample type	EV isolation method	Cohort size	Candidate biomarker(s)	Diagnostic/prognostic value	Independent validation	References
Plasma	Ultracentrifugation	10 AAA, 10 non-AAA controls	Ferritin light chain, HSP60, C-reactive protein, platelet factor 4 in exosomes; dermcidin in microparticles	Potential noninvasive diagnostic biomarkers for AAA	No	[83]
Activated platelet, AAA tissue (aneurysm wall and thrombus), plasma	Ultracentrifugation	478 AAA, 176 healthy controls	EV-carried ficolin-3	Early diagnosis, progression monitoring, and surgical risk prediction	No	[81]
Plasma; AAA tissue	Ultracentrifugation	21 AAA, 8 healthy controls	Exosomal miR-106a	Diagnostic and risk stratification biomarker	No	[67]
Serum	Chemical precipitation	35 AAA, 28 healthy controls	miR-122-5p and miR-483-5p in serum EVs	Specific liquid biomarkers for AAA and potential differential diagnosis	No	[79]
Human peripheral blood	Flow cytometry	12 non-endoleak AAA, 6 endoleak AAA	Activated endothelial-derived EVs (CD31^+^CD62P^+^)	Monitoring biomarker for post-EVAR endoleak	No	[84]
Plasma	Density-gradient ultracentrifugation	10 AAA, 14 non-AAA controls	Exosomal miR-122-5p	Noninvasive diagnostic biomarker and potential therapeutic target	No	[80]
Plasma	Chemical precipitation	In-house cohort: 10 AAA, 5 healthy controls; external cohort: 8 AAA, 4 healthy controls	IL-4, IL-6, MCP-1, neurturin, oncostatin-M in plasma EVs	Noninvasive diagnostic biomarkers and potential risk stratification panel	Yes, small external cohort	[82]

AAA, abdominal aortic aneurysm; EV, extracellular vesicle; HSP60, heat shock protein 60; miR, microRNA; IL, interleukin; MCP-1, monocyte chemoattractant protein-1; EVAR, endovascular aneurysm repair; CD31, cluster of differentiation 31; CD62P, cluster of differentiation 62P; non-AAA, non-abdominal aortic aneurysm.

Several limitations still need to be addressed. Many studies include small cohorts and lack independent validation, which increases the risk of cohort-specific or platform-specific findings [86]. Differences in EV isolation, characterization, analytical methods, and reporting standards also make results difficult to compare across studies. In addition, most AAA biomarker studies focus on blood-derived samples, such as plasma or serum, whereas more convenient sources for repeated sampling, especially urinary EVs, remain insufficiently explored [87]. More importantly, many candidate biomarkers have not been tested against clinically meaningful endpoints, such as aneurysm growth rate, rupture risk, or post-EVAR complications.

Future studies should therefore move from exploratory biomarker discovery toward clinically oriented validation. This requires larger multicenter cohorts, standardized EV workflows, prespecified endpoints, adjustment for key clinical covariates, and prospective longitudinal follow-up. Biomarkers should not only distinguish AAA patients from controls, but also provide information that can improve clinical decisions, such as identifying patients at higher risk of rapid expansion or complications. Therefore, the key challenge is no longer simply identifying additional candidate biomarkers, but converting promising EV-associated signals into reproducible, endpoint-linked, and clinically actionable tools.

**FIGURE 5:**
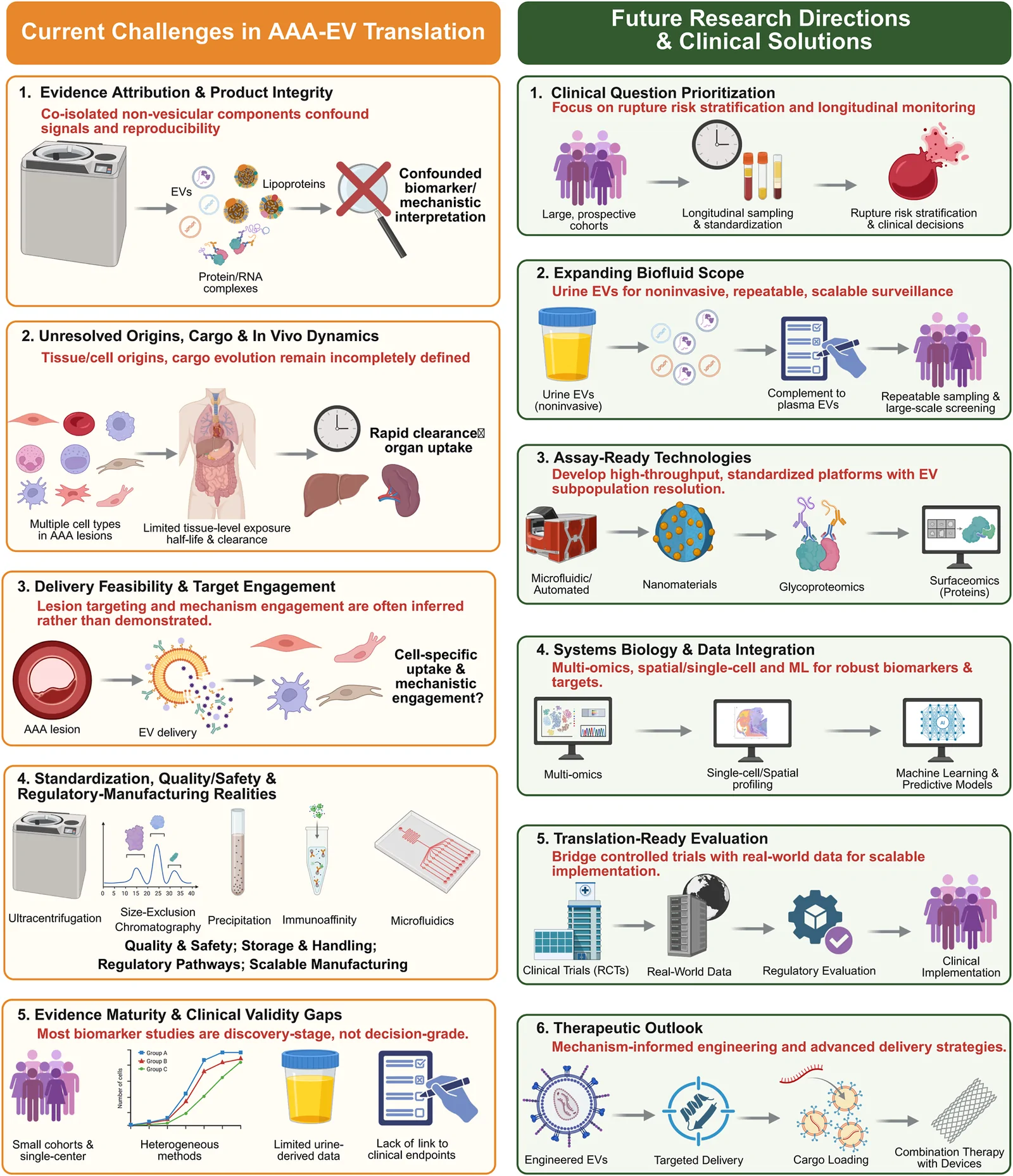
Current challenges and future research directions for AAA-EV translation. The diagram summarizes the major barriers limiting the clinical translation of extracellular vesicles (EVs) in abdominal aortic aneurysm (AAA) and proposes corresponding solutions. Current challenges include insufficient attribution of EV-specific signals due to co-isolated non-vesicular components such as lipoproteins and protein/RNA complexes, incomplete definition of EV tissue or cell origins, cargo evolution, biodistribution, half-life, and clearance, as well as limited direct evidence for lesion-specific delivery, cell-specific uptake, and mechanistic target engagement. Additional barriers include heterogeneous EV isolation and characterization methods, unresolved quality, safety, storage, regulatory, and manufacturing requirements, and the discovery-stage nature of most EV biomarker studies, which are often limited by small cohorts, single-center designs, heterogeneous workflows, limited urine-derived data, and weak linkage to clinical endpoints. Future directions include prioritizing rupture risk stratification and longitudinal monitoring, expanding urine EV analysis for noninvasive and repeatable surveillance, developing high-throughput assay-ready technologies with EV subpopulation resolution, integrating multi-omics, single-cell/spatial profiling, and machine learning to identify robust biomarkers and targets, and combining clinical trials with real-world data for translation-ready evaluation. Therapeutically, mechanism-informed EV engineering, targeted delivery, cargo loading, and combination strategies with devices may further support future AAA intervention. AAA, Abdominal Aortic Aneurysm; EVs, Extracellular Vesicles; ML, Machine Learning; RCTs, Randomized Controlled Trials.

Overall, the major challenge for EV-based AAA biomarkers is evidence maturity. Promising molecular signals must be converted into reproducible, externally validated, and clinically actionable tools (Figure 5). The same principle also applies to EV-based therapeutics: both diagnostic and therapeutic applications will require standardized methods, robust validation, and endpoint-aligned clinical evidence before they can move from proof-of-concept studies to real clinical use.

### Future research directions

Future AAA-EV research should be organized around clinical endpoints (rupture risk and longitudinal monitoring), assay-ready technology development, integrative data frameworks, and translation-ready evidence generation (Figure 5).

#### Clinical question prioritization: from AAA detection to rupture risk stratification and longitudinal monitoring

Aneurysm rupture is a fatal complication of AAA, causing approximately 200,000 deaths worldwide each year [88]. A paramount, unmet clinical need is the lack of biomarkers capable of accurately predicting AAA rupture risk. Future studies should be designed as large-scale, prospective cohorts to explore EV-based biomarkers that can stratify risk of rupture, ultimately guiding personalized surgical intervention decisions.

To maximize translational value, these studies should move beyond cross-sectional “AAA vs. control” comparisons and adopt longitudinal designs that link EV signatures to clinically actionable endpoints, with prespecified clinical covariates and standardized sampling schedules.

#### Expanding biofluid scope and enabling repeatable sampling: urine EVs as a practical route to scalable surveillance

Beyond blood-based biomarkers, the exploration of EVs in alternative biofluids, particularly urine, holds significant promise. Urine collection is a completely noninvasive, cost-effective, and suitable method for repeated sampling, making it an ideal source for large-scale screening and long-term monitoring [89]. Future research should focus on establishing the correlation between urinary EV cargo and the pathogenesis of AAA. The diagnostic and prognostic performance of urine EVs represents a critical metric for validating the clinical utility of EV-based biomarkers. Future large-scale, standardized, and outcome-oriented studies are warranted to evaluate their translational potential and facilitate clinical implementation rigorously.

In parallel, urine-based workflows can serve as a testbed for building scalable, standardized pipelines for pre-analytical handling and cross-site reproducibility, thereby accelerating downstream clinical validation.

#### From candidates to assays: technology development focused on throughput, standardization, and EV subpopulation resolution

Currently, most conventional methods struggle to meet the clinical requirements for throughput and standardization, and existing technologies also have limited ability to sort aneurysm-derived or specific-sized subpopulations of EVs. Thus, the development of novel EV isolation and analysis technologies is required. The latest advancements also focus on developing microfluidic, nanomaterial, or automated platforms to achieve a balance between speed, purity, and scalability [90, 91].

Moreover, the surface of EVs is rich in glycan structures (such as the glycocalyx), and the glycosylation patterns of EVs may serve as novel diagnostic biomarkers [92]. Additionally, the surface proteome of EVs contains unique transmembrane proteins and signaling molecules, which are associated with intercellular communication [93]. Surface proteins on EVs also determine binding capacity to recipient cells, thereby influencing pharmacokinetics and targeted delivery [94].

Therefore, advancements in glycoproteomic and surfaceomic technologies will provide new directions for research on biomarkers and therapeutic targets for AAA. Going forward, technology development should be aligned with clinical assay needs (automation, inter-laboratory reproducibility, and the ability to enrich disease-relevant EV subpopulations), rather than maximizing discovery depth alone.

#### Systems biology and data integration: multi-omics, single-cell/spatial profiling, and machine learning for robust biomarkers and targets

Integrated multiomics studies have provided new perspectives for understanding the complex pathological mechanisms of AAA [95]. Integration of transcriptomics, proteomics, and metabolomics data enables comprehensive assessment of biomarkers and potential targets for AAA. Concurrently, the development of bioinformatics tools for EV multiomics data will facilitate the extraction of clinically valuable biomarkers from big data. Through systems biology approaches, it will also help construct signaling networks to identify key nodes driving AAA progression, thereby improving diagnostic and therapeutic strategies for AAA [96].

Consistent with recent progress in aortic aneurysm research, future work should increasingly incorporate single-cell and spatial-omics frameworks to resolve lesion heterogeneity and identify previously unrecognized pathogenic cell subsets and communication axes that may be reflected in EV cargo. In addition, because single biomarkers often show limited specificity, building machine learning-based diagnostic and rupture-prediction models using EV multi-parameter features (proteins/ncRNAs/lipids) represents a promising direction, provided that models are trained and validated in large, multicenter populations.

#### Translation-ready evaluation: bridging controlled trials with real-world data and enabling scalable clinical implementation

In AAA research, integrating rigorously designed clinical trials with real-world data is important for enhancing the reliability of a study. Clinical trials provide strictly controlled conditions, while real-world data reflect the actual circumstances of patients in routine medical care [97]. Future efforts should focus on establishing standardized methods for collecting and analyzing real-world data and systematically documenting disease progression, treatment responses, and prognostic information [98]. By integrating real-world data with randomized controlled trial results, the clinical utility of EV biomarkers can be more comprehensively evaluated, providing evidence to support regulatory decisions and accelerating the translation and application of EV-related diagnostic products and treatment approaches.

To promote the clinical application of EVs in AAA, it is crucial to establish unified standardized guidelines, including standard operating procedures for the isolation, characterization, and quantification of EVs, thus ensuring the reproducibility of research and the reliability of results. Additionally, multicenter collaborative studies should be conducted to validate candidate biomarkers and therapeutic strategies, ultimately achieving the effective translation of EVs from the laboratory to clinical practice.

#### Therapeutic outlook: mechanism-informed engineering and device/material-enabled delivery without overextending current evidence

Looking ahead, the therapeutic development of EVs for AAA will likely depend on whether their biological advantages can be translated into more precise, reproducible, and clinically practical delivery systems. In this context, EV-based strategies should be considered alongside other therapeutic platforms under investigation for AAA, including liposomes, lipid nanoparticles, polymeric nanoparticles, and cell-based therapies [99–103]. Compared with synthetic nanoparticles, EVs more closely resemble endogenous delivery systems and may offer better biological compatibility, lower immunogenicity, and more active interactions with recipient cells through their native membrane structure and surface components [104]. However, liposomes, lipid nanoparticles, and polymeric nanoparticles currently retain important advantages in scalable manufacturing, batch consistency, quality control, and controllable cargo loading [105]. Cell-based therapies, by contrast, preserve broader adaptive and secretory functions but raise additional concerns regarding safety, persistence, and manufacturing complexity [106]. Because many of the therapeutic effects of mesenchymal stromal cell-based therapies are thought to be mediated largely through paracrine mechanisms, EVs may retain part of these biological benefits while reducing some cell-associated risks [107].

From this translational perspective, the future value of EVs in AAA may lie not in replacing all alternative platforms, but in occupying a complementary position between purely synthetic carriers and live-cell therapies. Native EVs lack inherent AAA-specific tropism, and their clinical application is limited by product heterogeneity, variable loading efficiency, and insufficient lesion targeting. These limitations define the central direction for future development: mechanism-informed engineering to improve payload definition and biological performance, together with device- or material-enabled strategies to enhance lesion targeting, vascular retention, and local delivery without overstating the maturity of current evidence. Anti-senescence modulation may represent one promising application within this broader framework [108–111]. Overall, future studies should prioritize translationally relevant optimization of EV design, dosing, delivery route, and formulation to more effectively translate the distinctive advantages of EVs can be more effectively converted into clinically credible AAA therapies.

### Conclusion

AAA remains a major age-associated vascular disease with limited options beyond surveillance and surgical repair, highlighting the need for better noninvasive biomarkers and disease-modifying therapies. This review summarizes growing evidence that EVs participate in AAA pathobiology as mediators of intercellular communication and may be leveraged as both diagnostic signals and cell-free therapeutic tools. Preclinical studies—predominantly using mesenchymal stem cell-derived and engineered EVs—suggest that EV-based interventions can modulate key processes such as macrophage-driven inflammation, vascular smooth muscle cell dysfunction, and matrix remodeling. To translate these advances into clinical practice, future work should prioritize decision-grade evidence through standardized workflows, rigorous EV characterization, lesion-level engagement readouts, and reproducible safety/quality frameworks in multicenter, endpoint-aligned studies. With these foundations, EV-based strategies may evolve from promising biological insights toward clinically credible tools for AAA risk stratification and aneurysm stabilization.
